# *In Vitro* Hepatoprotective and Human Gut Microbiota Modulation of Polysaccharide-Peptides in *Pleurotus citrinopileatus*

**DOI:** 10.3389/fcimb.2022.892049

**Published:** 2022-05-20

**Authors:** Yihua Huang, Yi Gao, Xionge Pi, Shuang Zhao, Wei Liu

**Affiliations:** ^1^ Disinfection Supply Center, Lishui Second People’s Hospital, Lishui, China; ^2^ Department of Stomatology, Beijing Xicheng District Health Care Center for Mothers and Children, Beijing, China; ^3^ Institute of Plant Protection and Microbiology, Zhejiang Academy of Agricultural Sciences, Hangzhou, China; ^4^ Institute of Agri-food Processing and Nutrition, Beijing Academy of Agriculture and Forestry Sciences, Beijing, China

**Keywords:** polysaccharide-peptide, *Pleurotus citrinopileatus*, hepatoprotection, gut microbiota, liver-gut axis

## Abstract

*Pleurotus citrinopileatus*, a golden oyster mushroom, is popular in Asia and has pharmacological functions. However, the effects of polysaccharide-peptides extracted from *Pleurotus citrinopileatus* and underlying mechanism on digestive systme have not yet been clarified. Here, we determined the composition of two polysaccharide-peptides (PSI and PSII) from *P. citrinopileatus* and investigated the protective effects of on hepatoprotective and gut microbiota. The results showed that PSI and PSII were made up of similar monosaccharide moieties, except for the varying ratios. Furthermore, PSI and PSII showed that they have the hepatoprotective effects and significantly increased the viabilities and cellular total superoxide dismutase activities increased significantly in HepG2 cells. Intracellular triglyceride content and extracellular alanine aminotransferase and aspartate transaminase contents markedly decreased following treatment with 40 and 50 μg/mL PSI and PSII, respectively. Moreover, PSI and PSII activated the adiponectin pathway and reduced lipid accumulation in liver cells. PSI and PSII elevated short-chain fatty acid concentrations, especially butyric and acetic acids. 16S rRNA gene sequencing analysis showed that PSI promoted the relative abundances of *Bifidobacteria*, *Lactobacillus*, *Faecalibacterium*, as well as *Prevotella* generas in the gut. PSII markedly suppressed the relative abundances of *Escherichia-Shigella* and *Bacteroides* generas. We speculate that the PSI and PSII play a role through liver-gut axis system. Polysaccharide-peptides metabolize by gut microbiota to produce short-chain fatty acids (SCFAs) and in turn influence liver functions.

## Introduction

Nonalcoholic fatty liver disease (NAFLD) is highly associated with chronic liver injury. In many countries, high-fat diets have increased the prevalence of NAFLD and lead to serious public health problems. NAFLD represents a spectrum of metabolic states that range from simple steatosis to non-alcoholic steatohepatitis, cirrhosis, hepatoma and fibrosis (Marchesini et al., 2001; Farrell and Larter, 2006). As a disorder, NAFLD is characterized by hypertriglyceridemia and abnormal hepatic fat accumulations, which are linked to obesity and insulin resistance (Svegliati-Baroni et al., 2006; Utzschneider and Kahn, 2006). Diet, exercise and antioxidants are currently the most effective treatments for NAFLD.

As a functional food, edible mushrooms of *Pleurotus* spp. are a potential natural source for drug candidates. Polysaccharides and polysaccharide-peptide complexes from *Pleurotus* spp. have anti-obesity (Sheng et al., 2019), antioxidant (Wu and Chen, 2017), antibacterial (Li and Shah, 2014), and antitumor effects (Ren et al., 2015) and have been shown to exhibit immunomodulatory activities, inducing macrophages to produce interleukins, nitric oxide, interferon-γ, and tumor necrosis factor (Cui et al., 2015). Moreover, polysaccharides from *Pleurotus* have been shown to have heptoprotective effects, including antihyperlipidemic activities. Indeed, these polysaccharides have preventive effects on high-fat diet-induced hyperlipidemia in mice, indicating potential beneficial effects on liver function (Zhang et al., 2017). Intracellular mycelial polysaccharides from *Pleurotus geesteranus* exhibit hepatoprotective effects against alcohol-induced acute alcoholic liver diseases, suggesting potential curative effects in alcoholic hepatitis (Song et al., 2018). However, the heptaoprotective effects of polysaccharide-peptides from *P. citrinopileatus* have not been fully evaluated.

The hepatointestinal system mediated nutrient digestion and absorption. NAFLD is associated with gut dysbiosis and changes in its metabolic functions (Boursier et al., 2016). Polysaccharide-peptides could be digest by gut microbiota to produce short-chain fatty acids (SCFAs) and in turn affect the liver functions. The study of the gut-liver axis can help us to understand the basic biology of NAFLD and identify the mechanisms between gut microbiota and liver damage (Tripathi et al., 2018).

In this study, we evaluated the hepatoprotective effects of two polysaccharide-peptides (PSI and PSII) extracted from *P. citrinopileatus* in a hepatoma cell model (HepG2 cells) of NAFLD. Furthermore, to assess the impacts of PSI and PSII on human gut microbiota, 16S rRNA sequencing techniques were used to explore the effects of PSI and PSII on gut microbiota by adult fermentation models *in vitro*, which is an effective tool for evaluating the impact of prebiotics on gut microbiota. Our findings elucidated on the use of PSI and PSII in improving human gut microbiota and develop new protective agents for the treatment of fatty liver and gut disease.

## Materials and Methods

### Materials and Regents

*P. citrinopileatus* fruiting bodies preserved at the Plant Protection Institute of Beijing Academy of Agricultural and Forestry Sciences (Beijing, China) were sun-dried and crushed to obtain fine powder. Standard monosaccharides (d-xylose, d-glucose, d-galacturonic acid, d-mannose, d-glucuronic acid, l-rhamnose, d-fructose, d-arabinose and d-galactose), oleic acid, DEAE-cellulose, 3-(4,5-dimethylthiazol-2-yl)-2,5-diphenyltetrazolium bromide (MTT), as well as palmitate were purchased from Sigma-Aldrich (USA). Superdex-200 column was acquired from the General Electric Company (GE, USA), and HepG2 cells were bought from the American Type Tissue Culture Collection (Manassas, VA, USA). Fetal bovine serum (FBS), Dulbecco’s modified Eagle’s minimum essential medium (DMEM), phosphate-buffered saline (PBS), penicillin, trypsin solution, and streptomycin were purchased from Invitrogen (USA). Protein, triglyceride (TG), alanine transaminase (ALT), aspartate transaminase (AST), and superoxide dismutase (SOD) assay kits were obtained from Nanjing Jiancheng Bioengineering Institute (Nanjing, Jiangsu Province, China). The rest of the chemicals as well as solvents were of analytical reagent grades and were obtained from Peking Chemical Co. (Beijing, China).

### Extraction and Purification of Polysaccharide-Peptides

The sun-dried fruiting bodies of *P. citrinopileatus* were placed in a high-speed universal crusher and repeatedly crushed four times for 20 s each. The crude polysaccharide-peptides were extracted thrice using the hot water method with a solid/liquid ratio of 1:50, at 90°C, and a 3 h extraction time. The obtained aqueous extracts were combined and concentrated using a rotary evaporator, and proteins in the concentrated solutions were removed by Sevag reagent (n-butanol and chloroform, 1:4 v:v ratio). Precipitation of the deproteinized solution was achieved by the addition of 100% ethanol (1:4 v:v ratio) at 25°C overnight, after which the polysaccharide-peptide extracts were acquired by centrifugation. Then, the polysaccharide-peptide extracts were dissolved in distilled water and applied to a DEAE-cellulose column (1 cm × 30 cm) that had been equilibrated with a 10 mM phosphate buffered solution (pH 7.0). Sequential elution of the column was done using 0, 0.2, and 1 M NaCl solution at a 1.5 mL/min flow rate. The adsorbed peak D2 and unadsorbed peak D1, with high carbohydrate levels as assessed by the phenol-sulfuric acid assay were collected. After being concentrated, to obtain bioactive PSI and PSII, the D1 and D2 fractions were applied to a Superdex-200 column equilibrated with ultrapure water using an AKTA Purifier (GE Healthcare).

### Analysis of PSI and PSII Monosaccharide Compositions, Fourier-Transform Infrared (FT-IR) Spectra, and Molecular Weights

Monosaccharide contents of PSI and PSII were analyzed by gas chromatography-mass spectrometry (GC-MS) (Yu et al., 2015). The IR spectras of PSI as well as PSII were evaluated by FT-IR (iS5 FTIR Spectrometer; Nicolet, USA) at 4000 to 400 cm^−1^. Molecular weights and homogeneity of PSI/PSII were determined by high-performance gel permeation chromatography (GPC) on TSK GMPWXL columns. Freeze-dried polysaccharide-peptides were analyzed by the Science Spectrum R&D Center (Shandong, China).

### Analysis of N-Terminal and Inner Amino Acid Sequences of PSI and PSII

Polysaccharide-peptide bands excised from sodium dodecyl sulfate-polyacrylamide (SDS-PAGE) gels were transferred to polyvinylidenedifluoride membranes followed by Coomassie brilliant blue R-250 staining. Stained bands were analyzed by the automated Edman degradation assay (Wang et al., 2018). Polysaccharide bands on SDS-PAGE gels was obtained and subjected to partial amino acid sequence analysis at Tsinghua University (Beijing, China). Using known sequences, sequence homology was searched in the BLAST/NCBI database.

### Analysis of the Cytotoxicity of Polysaccharides in Hepatocytes

Culture of HepG2 (hepatoma) cells was done in DMEM with 10% (v/v) FBS, 100 IU/mL penicillin and 100 mg/L streptomycin. Incubation at 37°C was done in a 5% (v/v) CO_2_ humid environment. Then, cells were seeded onto 96-well plates at 8 × 10^3^ cells/well followed by incubation for 12 h before the addition of PSI and PSII at 100, 200, 500, 800, or 1000 μg/mL concentrations. Incubation was then carried out for 72 more hours. Cytotoxicity was determined by MTT assays. Viability of PBS-treated control cells was set at 100%.

### Preparation of Double Factor-Induced Hepatocyte Injury

Free fatty acids (FFAs) and ethanol can induce hepatocyte injury. Palmitic and oleic acids were mixed (1:2, respectively) and used as FFAs (Garcia et al., 2011). For the hepatocyte injury model, HepG2 cell seeding in 96-well plates was done at a density of 8 × 10^3^ cells/well and subsequently incubated for 12 h after which FFAs and ethanol were added. Then, incubation was done for an additional 24 h, and MTT assays conducted to assess cell viabilities. PBS was used to replace FFAs and ethanol as the control.

### Protective Effects of PSI and PSII on Hepatocytes

To determine the protective effects of the polysaccharide-peptides, injured HepG2 cells (as described above) were treated for 48 h using varying PSI and PSII concentrations. Cell viabilities were then measured by MTT assays. Injured HepG2 cells were used as the negative control, and PBS without FFAs or ethanol was used as the positive control.

Optimal concentrations of PSI and PSII were used to analyze the mechanisms of action. Seeding of HepG2 cells was done in 6-well plates at 3 × 10^4^ cells/well, the injury model was induced, and cells were treated for 48 h using PSI and PSII. The cells as well as culture medium were then obtained to evaluate the protective and repairing abilities. The ALT as well as AST activities in the culture medium were measured using colorimetric assay kits. Quantification of cellular TG contents and SOD activities were done using commercial assay kits according to the manufacturers’ protocols. ALT, AST, and SOD results were expressed as U/mg protein. Oil-red O staining was done for histological analyses of cellular lipids.

### Gene Expression

Expressions of lipid metabolism-associated genes in the adiponectin pathway were assessed by quantitative real-time reverse transcription polymerase chain reaction (qRT-PCR). The TRIzol reagent (Invitrogen) was used for total RNA extraction from each sample. Reverse transcription of the extracted RNA was done in the presence of oligo (dT) using EasyScript First-Strand cDNA Synthesis SuperMix (Transgen, China), as instructed by the manufacturer. qRT-PCR was conducted according to the Maxima SYBR Green/ROX qRT-PCR Master Mix (Fermentas, USA) protocol using an ABI 7500 (Applied Biosystems, USA). In these experiments, GAPDH (glyceraldehyde 3-phosphate dehydrogenase) was used as the endogenous control. Gene-specific primer sets for mouse AdipoR2 (anti-adiponectin receptor 2), AMPK (AMP-activated protein kinase), CPTl (carnitine palmitoyltransferase 1), ACOX-1 (acyl-CoA Oxidase 1), PPARα (peroxisome proliferator–activated receptor α) were referred to previous studies (Spruiell et al., 2015; Han et al., 2019; Li et al., 2021). qRT-PCR conditions were: predenaturation for 5 min at 95°C and 40 cycles of 95°C for 30 s and for 1 min at 60°C. Relative expressions were calculated *via* the ΔΔCt method. Experiments were conducted in triplicates.

### *In Vitro* Batch Culture Fermentation

Preparation of the *in vitro* fermentation medium was done in 10 mL vials with 5 mL of anaerobic YCFA medium. YCFA medium consisted of (per 100 ml): 1 g casitone, 0.25 g yeast extract, 0.4 g NaHCO_3_, 0.1 g cysteine, 0.045 g K_2_ HPO_4_, 0.045 g KH_2_ PO_4_, 0.09 g NaCl, 0.009 g MgSO%.7H_2_ O, 0.009 g CaCl_2_, 0.1 mg resazurin, 1 mg haemin, 1 µg biotin, 1 µg cobalamin, 3 µg *p*-aminobenzoic acid, 5 µg folic acid and 15 µg pyridoxamine. Medium preparation was done in two different concentration gradients of PSI and PSII (40 µg/mL, 200 µg/mL) respectively, as the sole carbon source. Six healthy human volunteers (aged 22 - 42 years) from Hangzhou were enrolled in this study. They were fed on a normal Chinese diet, had no digestive ailments and had not been administered with any medications, including antibiotics, for >3 months before sample collections. Prior to inclusion in the study, volunteers were required to sign written informed consents. The Ethics Committee of Hangzhou center for diesease control and prevention (No. 202047) approved this study. The collection of fresh fecal samples was done in the morning. Then, preparation of fecal dilutions (10%) was done using the anaerobic phosphate buffer (PBS).

Suspension of 0.8 g Fresh fecal samples in 10 mL 0.1 mol/L anaerobic phosphate-buffered saline at pH 7.0 in an automatic fecal homogenizer was performed to obtain 10% (w/v) slurries. Then, 5 ml of the fecal suspensions were respectively inoculated into PSI and PSII medium as well as YCFA basal medium (control group). Batch fermentation was conducted *via* the inoculation of 1% fecal slurry into each vial followed by 24 h of incubation at 37°C.

### SCFAs Quantification

Crotonic acid (0.6464 g) was added into 2.5% (W:V) metaphosphoric acid solution (100 ml) to prepare the crotonic acid metaphosphoric acid solution. Then, 0.5 mL of the fermentation broth was added to 0.1 mL of the crotonic acid metaphosphoric acid followed by acidification at -20°C for more than 24 h prior to gas chromatography (GC) assay. Centrifugation of the fermentation broth was done for 3 min at 12, 000 rpm. Subsequently, SCFAs were detected in the supernatant *via* GC (GC, Shi-madzu, GC-2010 Plus, Japan). GC assays were done using a DB-FFAP column (Agilent Technologies, USA) and a H_2_ flame ionization detector. Acetic, propionic, isobutyric, butyric, pentanoic, isopentanoic and caproic acids were obtained from Sigma.

### 16S rRNA Gene Sequencing

Bacterial 16S rRNA gene V3–V4 hypervariable regions were amplified using 338F (5’-ACTCCTACGGGAGGCAGCA-3’) and 806R (5’-GGACTACHVGGG TWTCTAAT-3’) primers. The sequencing was performed on an Illumina MiSeq 2500 platform, and analyzed OEbiotech Co. Ltd. (Shanghai, China) for microbial diversity analysis. Representatively, one sequence was obtained from every Operational taxonomic unit (OTU), which were clustered at a similarity of 97% using Mothur software system. Taxonomic annotation of the OTUs was done using the RDP Classifier against SILVA database v. 128, at a 0.7 confidence threshold. Communal structure was analyzed at the phylum level and genus level based on the taxonomic information. LEfSe evaluations of the various groups were conducted and thresholds on the logarithmic score of linear discriminant analysis (LDA) set at 2.0. Deposition of the 16S sequencing data in the NCBI Sequence Read Archive (SRA) database was done under the accession number PRJNA751711.

### Statistical Analyses

Data are expressed as means ± SD of 3 replicates, and one-way ANOVA was used for statistical analyses by SPSS software. *p ≤*0.05 denoted statistical significance.

## Results

### Extraction and Purification of Polysaccharide-Peptides

**Figure 1:**
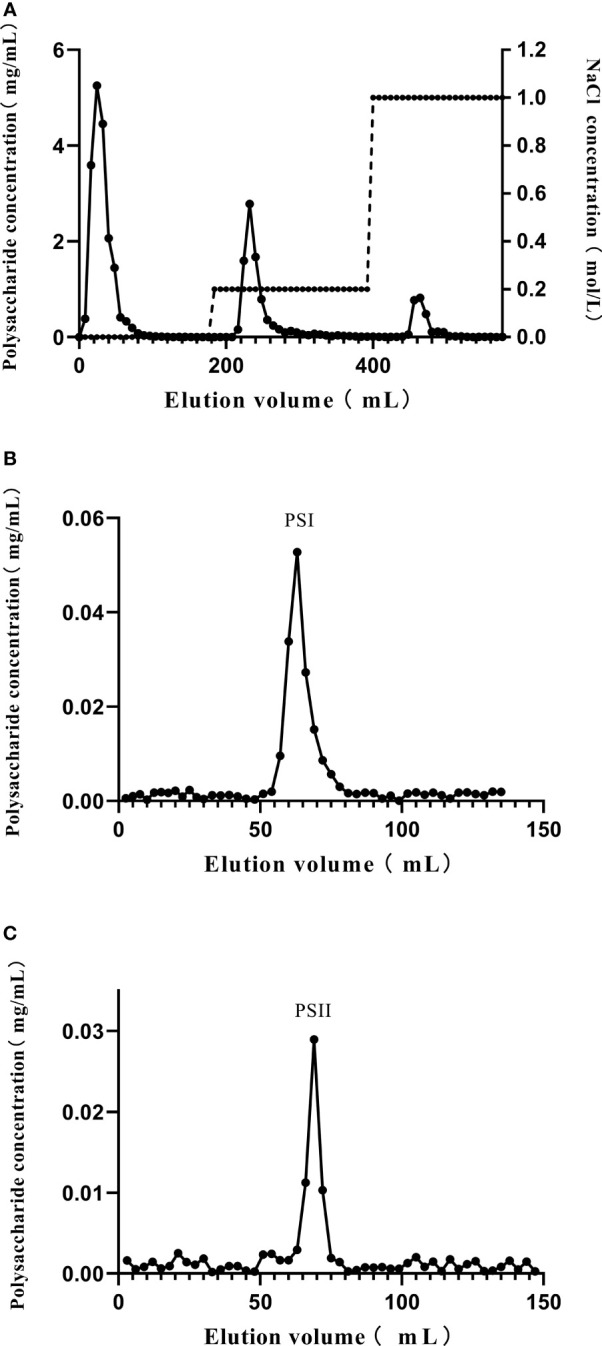
Purification of polysaccharide-peptides from *P. citrinopileatus*. **(A)** The crude polysaccharide-peptides were separated by chromatography on DEAE-cellulose columns. **(B)** D1 was further subjected to Sephadex 200 column chromatography. **(C)** D2 was further separated by Sephadex 200 column chromatography.

The crude polysaccharide-peptides were acquired by water extraction and alcohol precipitation from *P. citrinopileatus* fruiting bodies. Following removal of free proteins, purification of the crude polysaccharide-peptides was done in a DEAE-cellulose column. Three fractions eluted by phosphate buffer (10 mM, pH 7.0), phosphate buffer (10 mM, pH 7.0) with NaCl (0.2 M), and phosphate buffer (10 mM, pH 7.0) with 1 M NaCl were obtained (**Figure 1A**). D1 and D2, which had high polysaccharide content detected by the phenol-sulfuric acid assay, were then obtained, concentrated, dialyzed, and subjected to additional purification. The results showed that both D1 as well as D2 generated a single peak each (PSI and PSII, respectively; **Figures 1B, C**).

### Molecular Weight and Infrared Spectroscopy Analysis of PSI and PSII

The average molecular weight (Mw), number average molecular weight (Mn), and polydispersity (Mw/Mn) of PSI and PSII were evaluated by GPC. The Mw of PSI was 1.216 × 10^6^ Da, while its Mw/Mn value was 1.06. The Mw of PSII was 1.608 × 10^4^ Da, while its Mw/Mn value was 1.478 (**Table 1**).

**Table 1:** The average molecular weight (Mw), number average molecular weight (Mn), molecular weights, Z-average molecular weight (Mz) and the molecular weight of the highest peak (Mp) of PSI and PSII, as determined by GPC.

Molecular weight (Da)	PSI	PSII
Mn	1.147 × 10^6^	1.088 × 10^4^
Mw	1.216 × 10^6^	1.608 × 10^4^
Mz	1.231 × 10^6^	2.135 × 10^4^
Mp	4.308 × 10^5^	1.601 × 10^4^

**Figure 2:**
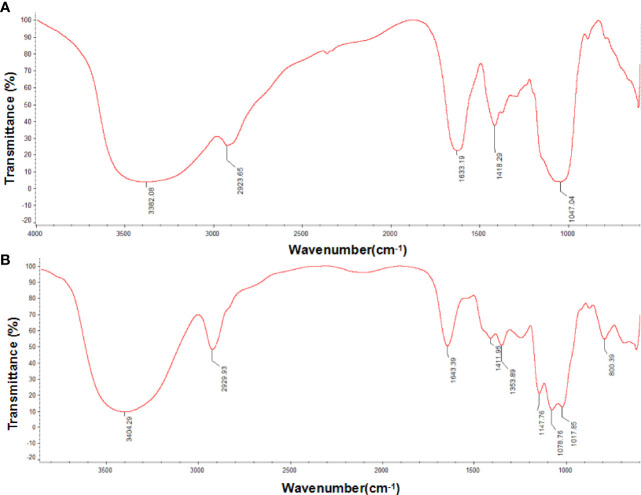
Fourier transform infrared (FT-IR) spectra of *P. citrinopileatus* polysaccharide-peptides. **(A)** PSI, **(B)** PSII.

Infrared absorption spectroscopy results are shown in **Figure 2**. PSI and PSII formed a broad peak about 3400 cm^-1^, representing the stretching vibration absorption peak of hydroxyl groups (Wang et al., 2017), and an absorption peak near 2929 cm^-1^, representing C-H (Dou et al., 2015), a characteristic peak of sugar. The peak near 1640 cm^-1^ indicated that both polysaccharides had C=O bonds (Liu et al., 2018), and the peak near 1017 cm^-1^ may be related to vibration of the ester carboxyl group (Lefsih et al., 2017). The absorption at 1411 cm^-1^ (**Figure 2A**) and 1418 cm^-1^ (**Figure 2B**) from O-H deformation indicated uronic acids presence. Regions at about 1078 cm^-1^ and 1047 cm^-1^ were representative of a galactan skeleton. Both PSI and PSII exhibited typical polysaccharide absorption peaks with characteristic groups of sugars, but with differences in chemical structure. The peaks of PSI at 1353^-1^ cm and 1259 cm^-1^ indicated the presence of an S=O bond, corresponding to an ester sulfate group. PSI had a specific band in the region of 1200–1000 cm^-1^, related to ring vibrations overlapping with stretching vibrations of the (C–OH) side groups as well as (C–O–C) glycosidic band vibrations.

### Monosaccharide Component Analysis

**Figure 3:**
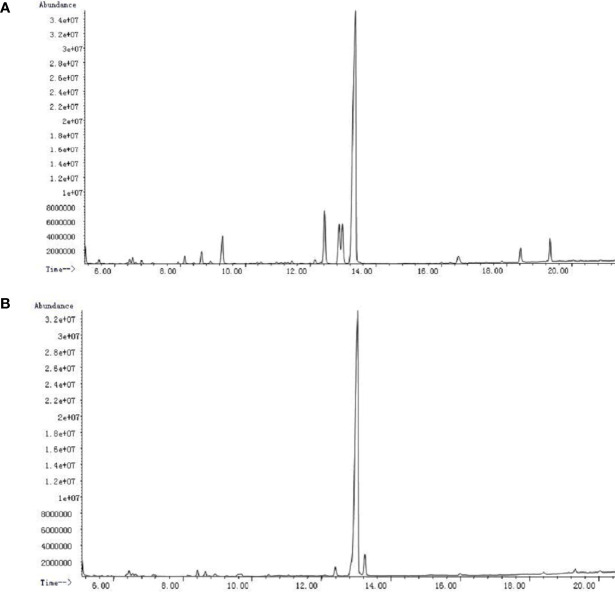
GC-MS chromatograms of *P. citrinopileatus* polysaccharide-peptides. **(A)** PSI, **(B)** PSII.

PSI and PSII were subjected to acid hydrolysis and analyzed by GC-MS after hydrolysis and silylation. PSI was made up of arabinose, mannose, glucose, and galactose at a molar ratio of 1:6.2:6.3:67.2 (**Figure 3A**). PSII was a heteropolysaccharide made up of xylose, glucose, and galactose at a molar ratio of 1:83.9:4.2 (**Figure 3B**).

### N-Terminal and Internal Amino Acid Sequences

N-terminal amino acid sequences of PSI and PSII were DLEQVVEGDW and KLSEGWERPP, respectively (**Supplemental Figure S1**). Evaluation of internal amino acid sequences revealed that the two peptide sequences of PSI, ITQSVLNIDR as well as VFQTNPNAFFR, were comparable to that of fruiting body lectin from *P. cornucopiae*. Moreover, the PSI sequences IQDKEGIPPDQQR, ISGLIYEETR, and KNGEILGGSWMVGAK were similar to those of ubiquitin, histone 4, and nucleoporin nup40 of *Lentinula edodes* (**Supplemental Table S1**). And three peptide sequences SSEREDLWQSTHVGHDEFSK, DGSLTGTYHSNVGEVPPTYHLSGR, and EDLWQSTHVGHDEFSK of PSII showed considerable homology with tamavidin-1 from *P. cornucopiae* (**Supplemental Table S2**). SYELPDGQVITIGNER and VAPEEHPVLLTEAPLNPK of PSII showed high similarity with actin-1 from *Hypsizygus marmoreus*.

### Cytotoxicity of PSI and PSII in HepG2 Cells

As shown in **Table 2**, cell viability was maintained at a high level (more than 90%) for all concentrations of PSI and PSII. Thus, PSI and PSII were not cytotoxic towards HepG2 cells and could be used as potential hepatoprotective drugs.

**Table 2:** The cytotoxicity of PSI and PSII in HepG2 cells.

Concentration (μg/mL)	Control	Cell viability (%)				
		100	200	500	800	1000
PSI	100	94.37 ± 3.62	100.40 ± 11.66	107.85 ± 5.28	108.71 ± 2.86	107.18 ± 7.24
PSII		95.18 ± 5.93	95.68 ± 2.87	106.05 ± 2.05	95.52 ± 4.25	94.93 ± 6.38

Data are presented as means ± SD (n = 3).

### Protective Roles of PSI and PSII *In Vitro*

**Figure 4:**
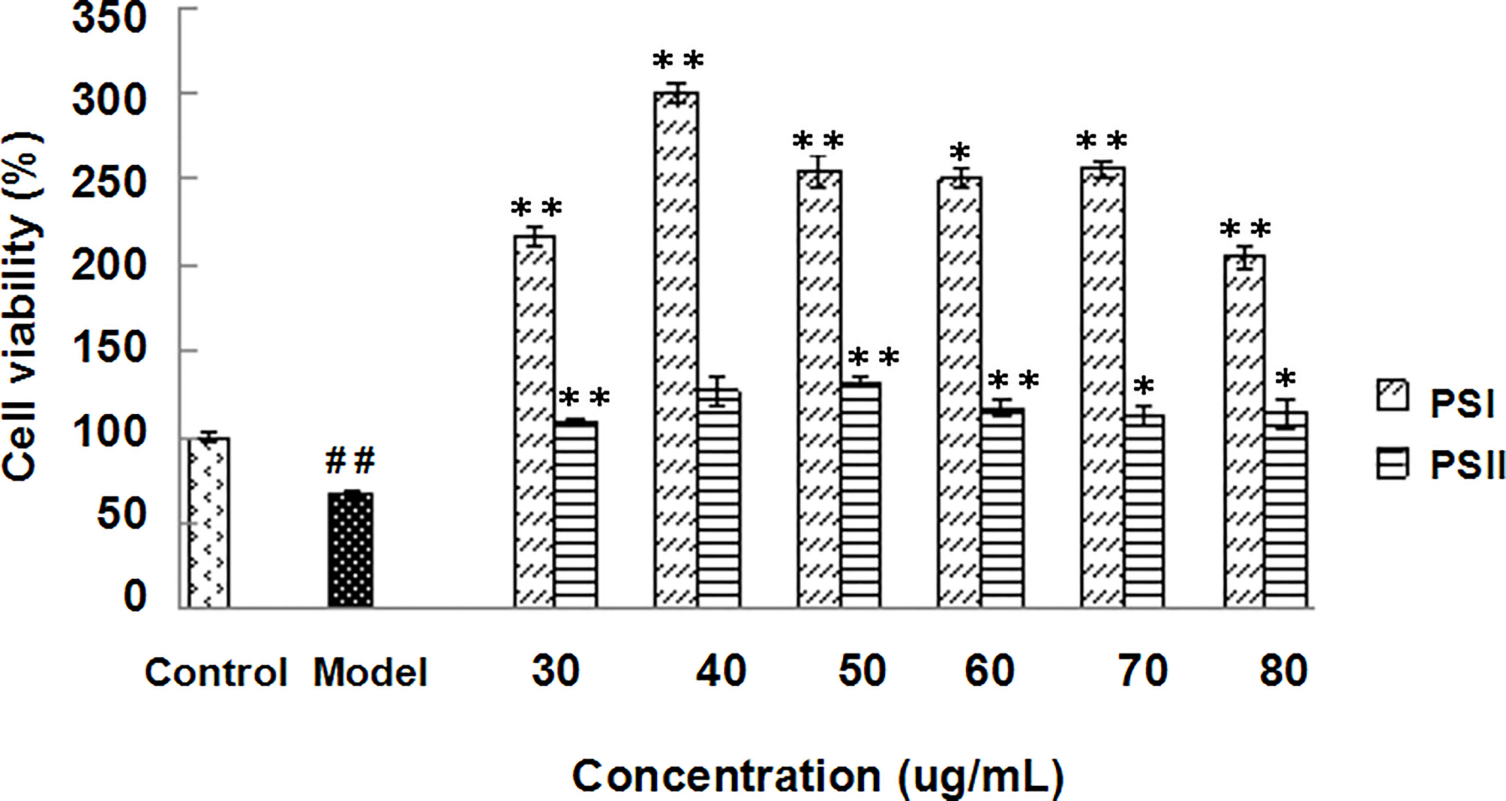
Hepatoprotective effects of PSI and PSII *in vitro*. ^##^ *P* < 0.01 relative to the control group, **P < 0.01 vs the model group, *P < 0.05 compared to the model group.

Induction of HepG2 cell injury models reduced cell viability to approximately 55–60%. MTT assays showed that all concentrations of PSI and PSII (30–80 μg/mL) increased the survival rates of injured cells. Compared with the model group, PSI and PSII increased cell viability up to 345.69% at 40 μg/mL and 96.15% at 50 μg/mL, respectively (**Figure 4**). The healing effects of PSI were better than those of PSII, and the survival rates of injured cells in the PSI and PSII treatment groups were both higher relative to the control group.

### Protective Mechanisms of PSI and PSII on the Liver Cellular Index

**Figure 5:**
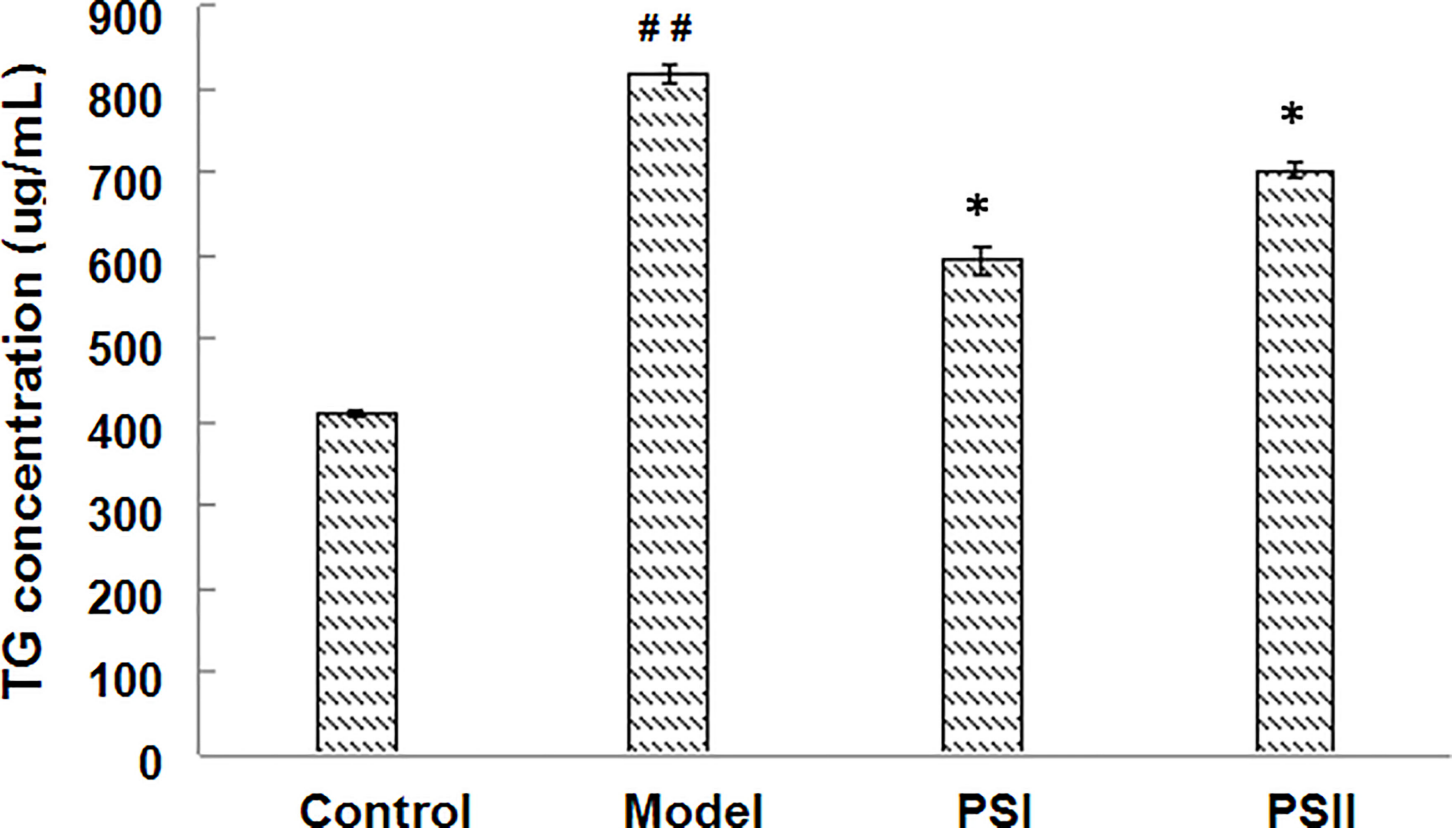
Effects of PSI and PSII on intracellular TG contents. ^##^ *P* < 0.01 relative to the control group, *P < 0.05 vs the model group.

**Figure 6:**
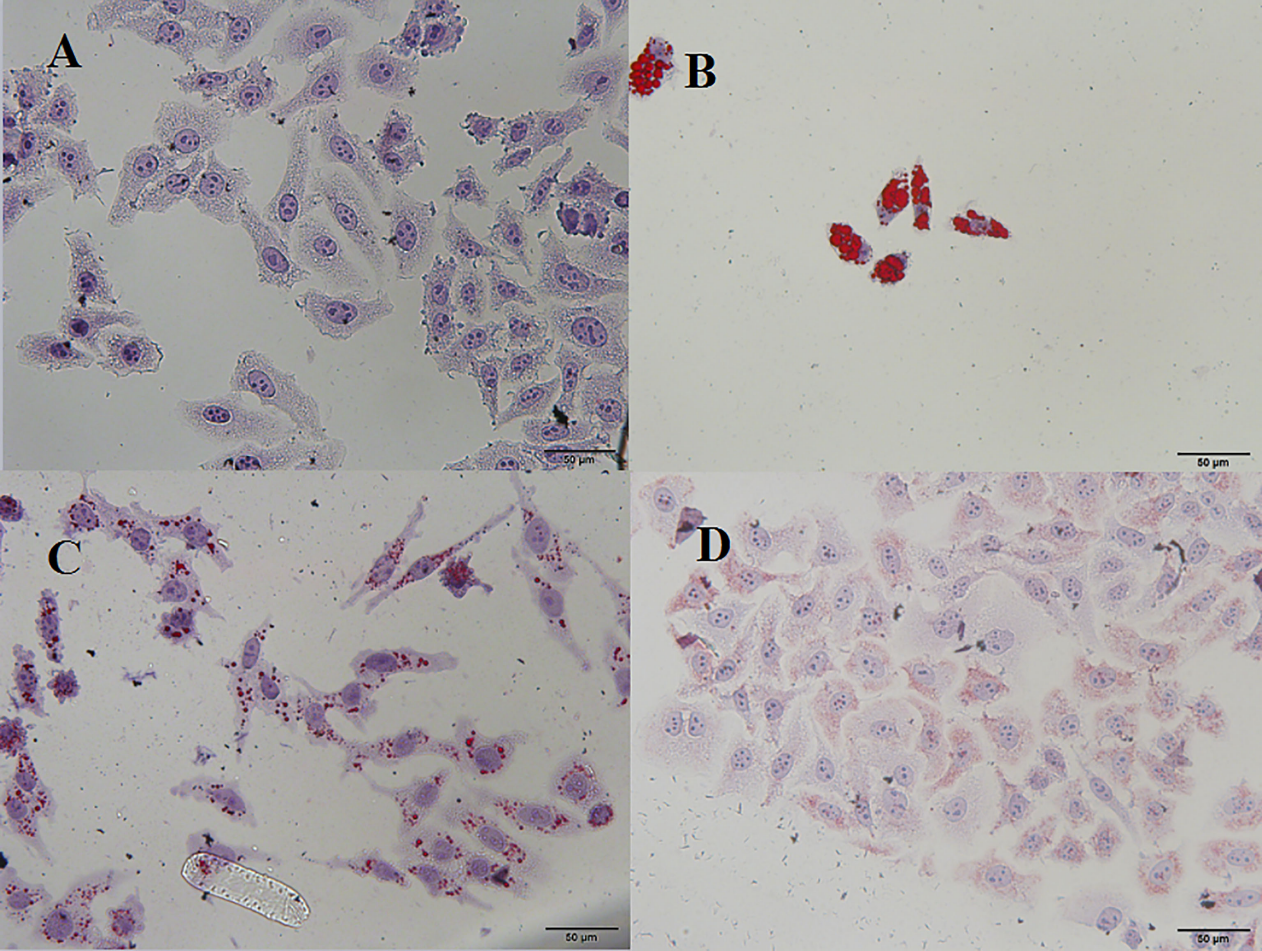
Microscopic view of Oil-red O staining in HepG2 cells. **(A)** Control, **(B)** model, **(C)** PSI, **(D)** PSII.

Excessive alcohol and fat intake can disrupt TG metabolism in hepatocytes (Wang et al., 2015). When synthesis rate exceeds anabolism rate, TGs accumulate in the liver, representing the main pathogenic factor of fatty liver disease (Yin et al., 2017). Accordingly, we evaluated the effects of PSI and PSII on intracellular TG content, total SOD activity, and extracellular AST and ALT levels. Intracellular TGs were significantly decreased by PSI and PSII treatment relative to model group (*P* < 0.05; **Figure 5**), suggesting that PSI and PSII blocked cellular lipid accumulation. These findings were verified by Oil-red O staining (**Figure 6**).

Moreover, PSI and PSII enhanced SOD activities in the cells by 66.35% and 21.71% (**Table 3**), respectively, indicating that the polysaccharide-peptides could increase the antioxidant activity of the cells. Extracellular ALT and AST levels could serve as indicators of liver cell status, with high values indicating liver damage (Kew, 2000). Additionally, PSI and PSII significantly reduced extracellular ALT and AST activities (*P* < 0.05; **Table 3**), indicating that PSI and PSII could block extracellular transaminase release, maintain cell integrity, and protect the liver.

**Table 3:** Protective effects of PSI and PSII on cellular indexes.

	Control group	Model group	PSI	PSII
T-SOD (U/mg)	12.67 ± 0.54*	7.37 ± 1.09	12.26 ± 0.94*	8.97 ± 0.37*
ALT (U/mg)	19983 ± 580*	27871 ± 71	22086 ± 96*	24825 ± 367*
AST (U/mg)	4846 ± 16*	13880 ± 43	8953 ± 93*	11662 ± 180*

Data are presented as means ± SD (n = 3). *P < 0.05 compared with the model group

### Therapeutic Mechanisms of PSI and PSII

**Figure 7:**
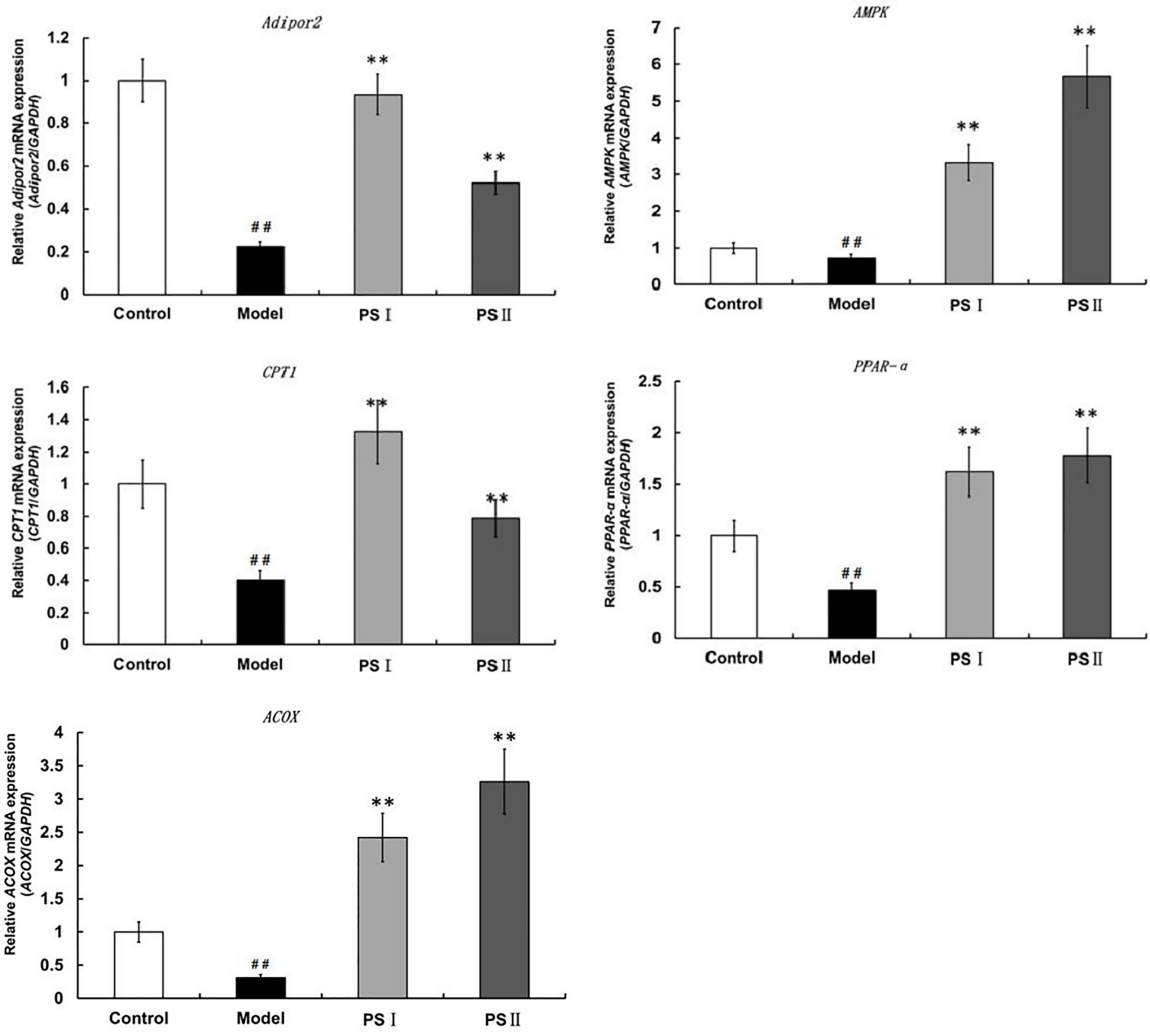
qRT-PCR assessment of PSI and PSII on gene expressions in adiponectin pathways. Data are presented as means ± SD (n = 3). ^##^ *P* < 0.01 relative to the control group, **P < 0.01 vs the model group.

We then analyzed changes in the adipogenic pathway in response to PSI and PSII by qRT-PCR (**Figure 7**). Notably, expression levels of genes encoding AdipoR2, AMP-activated protein kinase (AMPK), peroxisome proliferator-activated receptor α (PPARα), carnitine palmitoyltransferase 1 (CPTI), and peroxisomal acyl-coenzyme A oxidase 1 (ACOX1) were markedly suppressed in the model group. Additionally, levels of TG in the model group were significantly increased. In contrast, treatment with PSI and PSII reduced hepatic lipogenesis by increasing *AdipoR2*, *AMPK*, *CPTl*, *PPARα*, and *ACOX1* expression in adipocytes to stimulate adiponectin secretion as well as activate the FFA metabolic pathway, thereby promoting triglyceride metabolism and reducing lipid accumulation.

### Effects of PSI and PSII on SCFAs Production

**Figure 8:**
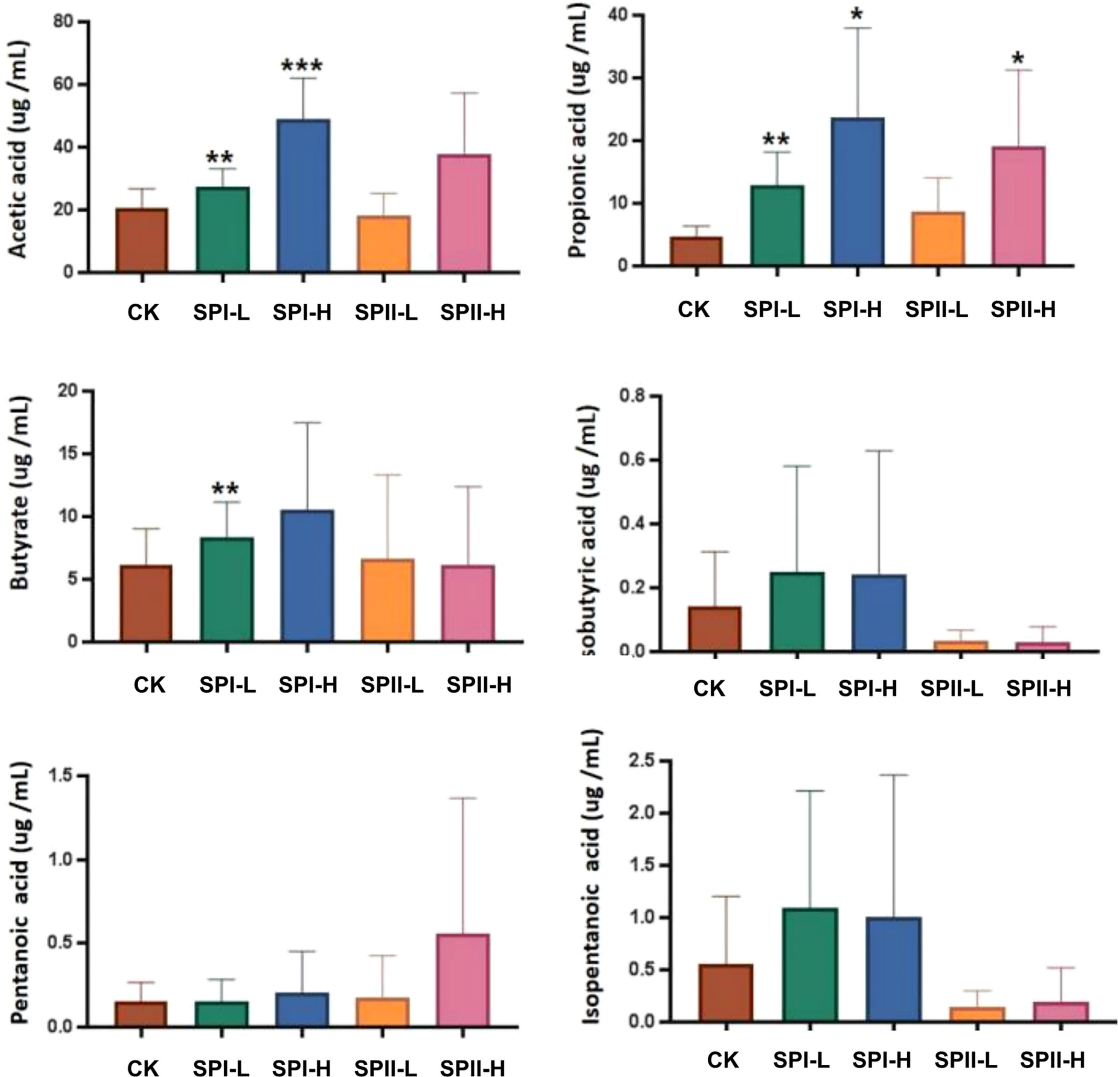
Short-chain fatty acids (SCFAs) levels in fermentation broth after 24 h of fermentation. (G1) 40 µg/mL PSI, (G2) 200 µg/mL PSI, (H1) 40 ug/mL PSII, (H2) 200 ug/mL PSII. *P < 0.05, **P < 0.01 or ***P < 0.001 relative to control group.

Productions of SCFAs were achieved *via* fermentation of PSI and PSII in human fecal samples (**Figure 8**). Relative to the control group, propionate, acetate and butyrate concentrations were significantly higher in the PSI group. However, isobutyric acid and isopentanoic acid concentrations in the PSII group were low relative to the control group.

### Effect of PSI and PSII on the Bacterial Community

**Figure 9:**
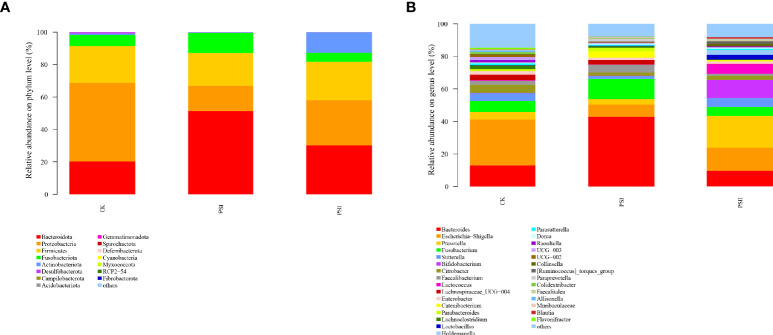
Effects of PSI and PSII on the gut microbiota. **(A)** Bacterial phyla abundance; **(B)** Microbial communities on the genus level after various treatments. The ordinate denotes the species name, color gradient denotes species proportions, while the right side of the figure represents the value represented by the color gradient.

Based on 16S rRNA sequencing, *Bacteroidetes*, *Proteobacteria*, *Firmicutes*, *Fusobacteriota* and *Actinobacteria* were found to be the abundant phyla in test samples (**Figure 9A**). After 24 h of fermentation with PSI and PSII, the increases in abundances of Bacteroidota and *Fusobacteriot*a for PSI and *Bacteroidota* and *Actinobacteria* and for PSII, respectively were significant. However, *Proteobacteria* enrichment in PSI and PSII groups were markedly suppressed relative to the control group.

The relative abundances of *Bacteroides, Fusobacterium, Faecalibacterium, Catenibacterium, Blautia* at the genus level were markedly higher in the PSI groups relative to the control group, and the abundance of *Escherichia-Shigella, Sutterella* and *Flavonifractor* were markedly low in the PSI group relative to the control (**Figure 9B**). In the PSII groups, the abundance of *Prevotella*, *Sutterella*, *Bifidobacterium*, *Lactococcus*, *Lactobacillus*, *Holdemanella, Catenibacterium* and *Blautia* were significantly high at genus levels, and the relative abundance of *Escherichia-Shigella, Faecalibacterium, Lachnospiraceae*_UCG 004, *Lachnoclostridium* and *Flavonifractor* was significantly lower relative to the control group.

**Figure 10:**
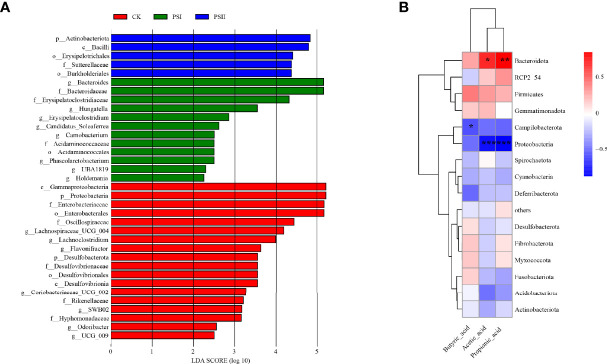
Diagram of the linear discriminant (LDA) score and correlation analysis. **(A)** LDA score between the control and polysaccharide-peptieds groups (PSI, PSII), with a 2.0 score threshold. Diagram of the linear discriminant analysis (LDA) score between the control and polysaccharide-peptides groups (PSI, PSII), with a 2.0 score threshold. **(B)** Correlations between gut microbiota and metabolites SCFA factor fermented by PSI. The X axis represents SCFA species while the Y axis denotes the species. Color depth denotes R value size, while the legend denotes color intervals for various R values. *P < 0.05, **P < 0.01, ***P < 0.001.

Identification of bacterial taxa with significant differences in abundance between polysaccharide-peptides groups and the control groups was done by the linear discriminant analysis (LDA) effect size (LEfSe) method. Wilcoxon rank-sum test revealed that PSI enhanced the proliferation of *Bacteroides*, *Erysipelatoclostridiaceae*, *Hungatella*, *Carnobacterium*, *Acidaminococcaceae and Phascolarctobacterium*, while *Proteobacteria*, *Enterobacteriaceae, Oscillospiraceae*, *Desulfovibrionaceae*, *Lachnoclostridium*, *Flavonifractor*, *Odoribacter and Coriobacteriaceae* were markedly enriched in PSII group (**Figure 10A**). These results showed that PSI and PSII can regulate gut microbiota and promote probiotic proliferation.

### Correlation Between Gut Microbiota and Metabolites Factors

Previous experiments showed that PSI had better effects on SCFAs production. Therefore, we studied the correlation between the production of SCFAs metabolites and the gut microbiota community of PSI. Acetic acid and propionic acid content were positively associated with the abundance *Bacteroidota* (**Figure 10B**). However, their contents were negatively associated with *Proteobacteri*a abundance. Butyric acid levels negatively correlated with *Campilobacterota* abundance.

## Discussion

NAFLD, a prevalent chronic liver disease, can cause several other diseases (Cohen et al., 2011). Some research groups have confirmed the protective effects of a polysaccharide-enriched fraction from *Pleurotus* sp. *in a* model of liver injury. Polysaccharides from *P. geesteranus* show antioxidant and hepatoprotective effects for preventing alcoholic liver diseases (Song et al., 2018). Moreover, mycelia zinc polysaccharides from *P. djamor* prevent CCl_4_-induced acute liver damage, and alleviate liver as well as kidney injury in streptozocin-induced diabetic mice (Zhang et al., 2015).

The clinical diagnosis of NAFLD is based on elevation of serum ALT as well as AST, biochemical biomarkers of liver injury (Song et al., 2014; Liang et al., 2015). Herein, we investigated the antihyperlipidemic and hepatoprotective effects of PSI and PSII in a hepatocyte injury model. The results showed that PSI and PSII significantly reversed elevations in ALT/AST levels and increased cellular SOD activity and cell viability in HepG2 cells. SOD is a key player regulating cellular defense against reactive oxygen species. Moreover, PSI and PSII treatment markedly reduced TG contents, supporting the therapeutic effects of PSI and PSII in fatty liver.

Some groups reported the immune-promoting effects of polysaccharide-peptide and polysaccharide-protein complex obtained from mushrooms (Wang et al., 1996; Maruyama and Ikekawa, 2005). Li et al. isolated a polysaccharide-peptide complex from *Pleurotus abalonus* and found that it exhibited anti-proliferative, hypoglycaemic and antioxidant activities (Li et al., 2012). In this study, the IQDKEGIPPDQQR and KNGEILGGSWMVGAK sequences of PSI were similar to those of ubiquitin and nup40. nup40 has mitotic spindle checkpoint functions and inhibits cell cycle progression by binding to components of the ubiquitin-conjugating system (Chen et al., 2004). This may explain the effects of PSI on promoting cell viability.

FFAs stimulate hepatic TG synthesis and cause hepatic lipotoxicity as well as inflammation, thereby promoting NAFLD pathogenesis. Disruption of hepatocyte FFA metabolism and *de novo* FFA synthesis are involved in establishment of NAFLD. In hepatocytes, PPAR-α is a central regulator of TG and fatty acid metabolism (Aoyama et al., 1998; Kamijo et al., 2007) Additionally, ACOX1 catalyzes first as well as rate-limiting enzyme in fatty acid β-oxidation pathway of very-long-chain fatty acids in peroxisomes, which can be activated by PPARα to stimulate hepatic fatty acid oxidation (Fan et al., 1998). Gene expression assays revealed that PSI and PSII up-regulated *PPARα* and *ACOX1* as well as the downstream target gene *CPT1*, which is involved in peroxisomal as well as mitochondrial oxidation of fatty acids (Pathil et al., 2015). Thus, PSI and PSII stimulated FFA oxidation, which resulted in burning of excess energy in the liver.

Adiponectin, which is secreted by adipocytes, promotes the oxidation of fatty acids and modulates lipid metabolism by mediating the expressions of hepatic genes critical for lipid metabolism (Liu et al., 2012). The AMPK pathway can also be activated adiponectin. AdipoR2 is an adiponectin receptor that regulates lipid metabolism, fatty-acid oxidation, and adiponectin-induced biological functions (Kadowaki and Yamauchi, 2005; Ghadge et al., 2018) Therefore, adiponectin and AdipoR2 are potential therapeutic targets to combat NAFLD. In this study, we found that that PSI and PSII increased *AMPK* and *AdipoR2* mRNA expression, implying that PSI and PSII activated adiponectin-related pathways and accelerated lipid metabolism.

SCFAs, major by-products of microbial metabolism in the liver, play important roles in maintaining large intestine functions and colon epithelial cells. Many prebiotics can increase the content of SCFAs (Liu et al., 2020; Zhao et al., 2021), which could promote the human health *via* an indirect effects. SCFAs can mediated the gut microbiota modulation of host physiological as well as pathological processes (Koh et al., 2016). This study showed that PSI fermentation markedly elevated acetic, propionic, and butyric acids concentrations. Acetic acid, butyric acid and propionic acid are important mediators of fermented dietary fibers on metabolism (den Besten et al., 2013). Butyrate and propionate could suppress lipolysis as well as *de novo* lipogenesis, thereby protecting against obesity development (Lin et al., 2012; Heimann et al., 2015). Acetic acid can reduce appetite and intestinal inflammation, and inhibits human fat decomposition (Duseja and Chawla, 2014; Gangarapu et al., 2014). It indicates that PSI enhances the proliferation of bacteria that produce acetic acid, butyric acid and propionic acid. We speculate that the PSI and PSII play a role through liver-gut axis system. Intestinal allows microbial metabolites and microbial-associated molecular patterns to translocate to the liver (Tripathi et al., 2018; Ciaula et al., 2020). Polysaccharide-peptides were metabolized by gut microbiota to produce SCFAs and in turn influence liver functions. The study of the gut-liver axis can help us to understand the basic biology of NAFLD and identify the mechanisms between gut microbiota and liver damage, which offers an opportunity for interventions during liver disease.

The microbiota is required to maintain hepatic homeostasis. The changes in the gut microbiota have disclosed the interaction with the pathogenesis of NAFLD. The severity of NAFLD is associated with dysbiosis of the intestinal (Marra and Svegliati-Baroni, 2018). On account of gut microbiota is linked to NAFLD, we evaluated the effects of PSI and PSII on human intestinal microflora structure. Analysis of bacterial community composition showed that PSI and PSII promoted the proliferation of probiotics and inhibit the harmful bacteria, such as *Escherichia-Shigella*. There was a markedly elevated abundance of *Phascolarctobacterium*, *Bacteroides*, *Fusobacterium*, *Faecalibacterium*, *Catenibacterium*, *Blautia* at the genus level in the PSI groups. *Phascolarctobacterium* is a SCFAs producer, including acetic acid and propionic acid (Wu et al., 2017). Accumulated evidences showed that *Phascolarctobacterium faecium* has beneficial effects on the NAFLD rat model (Panasevich et al., 2016). *Fusobacteriota* metabolized carbohydrates into butyrate which has benefits to the host (Zhang et al., 2021). In the PSII groups, there were markedly higher abundances of *Bifidobacterium*, *Lactococcus*, *Lactobacillus*, *Desulfovibrionaceae*, *Lachnospiraceae*, *Odoribacter*, *Coriobacteriaceae* and *Blautia* at the genus level. *Bifidobacterium* and *Lactobacillus* are well-known probiotics. *Blautia* is also a potential probiotic (Liu et al., 2021). *Coriobacteriaceae* can metabolize cholesterol-derived metabolites (McGavigan et al., 2017). The family of *Lachnospiracea*e produces short-chain fatty acids, and previous *s*tudy corroborated *Lachnospiraceae* in attenuating colitis and obesity (Guo et al., 2020). *Odoribacter splanchnicus* induced Th17 cell activated and protected mice from colitis and colorectal cancer (Xing et al., 2021). Evidence suggests that PSI and PSII can be used as potential prebiotics to regulate gut microbiota.

## Conclusion

In this study, we purified and characterized PSI and PSII from *P. citrinopileatus*. These compounds exhibited hepatoprotective effects in injured HepG2 cells by increasing the survival rates of injured cells, reducing the accumulation of intracellular TGs, elevating the intracellular activity of SOD, decreasing extracellular transaminase release, and maintaining cell integrity. These results suggested that PSI and PSII exert potent antioxidant and hepatoprotective activities by regulating the expression of hepatic genes. On the other hand, PSI and PSII supplementation to an *in vitro* fermentation model affected human gut microbiota richness as well as diversity. PSII enhanced the abundance of *Oscillospiraceae*, *Lachnospiraceae*, *Lachnoclostridium*, *Flavonifractor*, *Desulfobacterota*, *Desulfovibrionaceae*, *Coriobacteriaceae*, *Rikenellaceae, Odoribacter*. PSI and PSII decrease the abundance of *Escherichia-Shigella* genera. Moreover, PSI enhanced the metabolism of acetic, propionic, as well as butyric acids in bacteria, which resulted in elevated concentrations of SCFAs. These SCFAs exert an indirect effect on intestinal microbiota and liver functions. PSI and PSII might play a role through liver-gut axis system. These findings provided important insights into the potential applications of PSI and PSII in ameliorating symptoms of liver disease and gut microbiota modulation.

## Data Availability Statement

The datasets presented in this study can be found in online repositories. The names of the repository/repositories and accession number(s) can be found below: https://www.ncbi.nlm.nih.gov/genbank/, PRJNA751711.

## Ethics Statement

The studies involving human participants were reviewed and approved by The Ethics Committee of Hangzhou center for disease control and prevention. The patients/participants provided their written informed consent to participate in this study.

## Author Contributions

WL and SZ designed the study and all experiments. YH and YG carried out the assays. YG and XP analyzed the data. YH drafted this manuscript. WL and SZ revised the manuscript. All authors have read and approved the submitted version.

## Funding

This work was supported by the Collaborative Innovation Center of Beijing Academy of Agricultural and Forestry Sciences (Grant No. KJCX201915), Beijing Academy of Agriculture and Forestry Science (Grant No. KJCX20200208), Zhejiang Public Welfare Project (Grant No. LGN22C030008), and Hangzhou Agricultural and Society Development Project (Grant No. 202004A20).

## Conflict of Interest

The authors declare that the research was conducted in the absence of any commercial or financial relationships that could be construed as a potential conflict of interest.

## Publisher’s Note

All claims expressed in this article are solely those of the authors and do not necessarily represent those of their affiliated organizations, or those of the publisher, the editors and the reviewers. Any product that may be evaluated in this article, or claim that may be made by its manufacturer, is not guaranteed or endorsed by the publisher.
