# Supplementary material for: In Vitro Hepatoprotective and Human Gut Microbiota Modulation of Polysaccharide-Peptides in Pleurotus citrinopileatus
Source: Front Cell Infect Microbiol. 2022 May 20;12:892049. doi: 10.3389/fcimb.2022.892049 (PMC9165600; doi:10.3389/fcimb.2022.892049)
Supplement: Supplementary file 1 [file DataSheet_1.zip › Table 1.DOCX]

Supplementary Table 1. The descriptions of prediction proteins sequences of PSI fragment from *Pleurotus citrinopileatus.*

| Accession | Description | Score | Coverage | # Proteins | # Unique Peptides | # Peptides | # PSMs | # AAs | MW [kDa] | calc. pI |
| --- | --- | --- | --- | --- | --- | --- | --- | --- | --- | --- |
| A0A151V7G0 | Actin-1 OS=Hypsizygus marmoreus OX=39966 GN=ACT1 PE=3 SV=1 - [A0A151V7G0_HYPMA] | 52.53 | 14.67 | 11 | 4 | 4 | 18 | 375 | 41.6 | 5.48 |
| Q96WQ6 | Fruit body lectin OS=Pleurotus cornucopiae OX=5321 GN=PCL-F1 PE=4 SV=1 - [Q96WQ6_PLECO] | 11.63 | 14.58 | 2 | 2 | 2 | 4 | 144 | 16.2 | 7.34 |
| A0A1Q3EQ96 | Ubiquitin OS=Lentinula edodes OX=5353 GN=LENED_011525 PE=4 SV=1 - [A0A1Q3EQ96_LENED] | 7.28 | 17.06 | 10 | 1 | 1 | 3 | 381 | 42.7 | 7.58 |
| A0A1Q3DXK4 | Nucleoporin nup40 OS=Lentinula edodes OX=5353 GN=LENED_001225 PE=4 SV=1 - [A0A1Q3DXK4_LENED] | 3.14 | 4.67 | 1 | 1 | 1 | 1 | 321 | 34.9 | 6.74 |
| A0A151W8W4 | Ribosomal RNA-processing protein 12 OS=Hypsizygus marmoreus OX=39966 GN=RRP12 PE=4 SV=1 - [A0A151W8W4_HYPMA] | 2.85 | 0.81 | 1 | 1 | 1 | 1 | 1234 | 134.3 | 8.24 |
| A0A1Q3EJ42 | Histone H4 OS=Lentinula edodes OX=5353 GN=LENED_009215 PE=3 SV=1 - [A0A1Q3EJ42_LENED] | 2.75 | 10.99 | 8 | 1 | 1 | 1 | 91 | 9.9 | 11.41 |
| A0A1Q3ENT5 | ATP synthase subunit beta OS=Lentinula edodes OX=5353 GN=LENED_010958 PE=3 SV=1 - [A0A1Q3ENT5_LENED] | 2.52 | 2.06 | 3 | 1 | 1 | 1 | 533 | 57.2 | 5.94 |
| A0A1Q3ETC9 | 2-cysteine peroxiredoxin OS=Lentinula edodes OX=5353 GN=LENED_012718 PE=4 SV=1 - [A0A1Q3ETC9_LENED] | 2.38 | 5.58 | 3 | 1 | 1 | 1 | 197 | 21.9 | 4.88 |
| A0A1Q3EFH4 | ATP synthase F1 alpha subunit OS=Lentinula edodes OX=5353 GN=LENED_007868 PE=4 SV=1 - [A0A1Q3EFH4_LENED] | 1.69 | 3.57 | 3 | 1 | 1 | 1 | 364 | 39.9 | 8.29 |
| A0A1Q3ETM3 | Uncharacterized protein OS=Lentinula edodes OX=5353 GN=LENED_012865 PE=4 SV=1 - [A0A1Q3ETM3_LENED] | 0.00 | 3.11 | 1 | 1 | 1 | 1 | 193 | 21.0 | 8.12 |
| A0A151W569 | Uncharacterized protein OS=Hypsizygus marmoreus OX=39966 GN=Hypma_03088 PE=4 SV=1 - [A0A151W569_HYPMA] | 0.00 | 7.58 | 1 | 1 | 1 | 1 | 330 | 37.2 | 7.20 |

Supplementary Table 2. The descriptions of prediction proteins sequences of PSII fragment from *Pleurotus citrinopileatus.*

| Accession | Description | Score | Coverage | # Proteins | # Unique Peptides | # Peptides | # PSMs | # AAs | MW [kDa] | calc. pI |
| --- | --- | --- | --- | --- | --- | --- | --- | --- | --- | --- |
| B9A0T6 | Tamavidin1 OS=Pleurotus cornucopiae OX=5321 GN=tam1 PE=2 SV=1 - [B9A0T6_PLECO] | 161.91 | 30.77 | 1 | 3 | 3 | 46 | 143 | 16.0 | 6.68 |
| B9A0T7 | Tamavidin2 OS=Pleurotus cornucopiae OX=5321 GN=tam2 PE=1 SV=1 - [B9A0T7_PLECO] | 47.42 | 20.57 | 1 | 2 | 2 | 13 | 141 | 15.5 | 7.43 |
| A0A1Q3EQ96 | Ubiquitin OS=Lentinula edodes OX=5353 GN=LENED_011525 PE=4 SV=1 - [A0A1Q3EQ96_LENED] | 21.31 | 17.06 | 10 | 1 | 1 | 7 | 381 | 42.7 | 7.58 |
| A0A151V7G0 | Actin-1 OS=Hypsizygus marmoreus OX=39966 GN=ACT1 PE=3 SV=1 - [A0A151V7G0_HYPMA] | 19.56 | 9.07 | 10 | 2 | 2 | 6 | 375 | 41.6 | 5.48 |
| A0A1Q3ENT5 | ATP synthase subunit beta OS=Lentinula edodes OX=5353 GN=LENED_010958 PE=3 SV=1 - [A0A1Q3ENT5_LENED] | 13.48 | 7.88 | 3 | 3 | 3 | 4 | 533 | 57.2 | 5.94 |
| A0A1Q3EJ42 | Histone H4 OS=Lentinula edodes OX=5353 GN=LENED_009215 PE=3 SV=1 - [A0A1Q3EJ42_LENED] | 11.10 | 10.99 | 8 | 1 | 1 | 4 | 91 | 9.9 | 11.41 |
| P83467 | Ostreolysin A6 OS=Pleurotus ostreatus OX=5322 GN=OlyA6 PE=1 SV=2 - [OLYA6_PLEOS] | 8.74 | 15.22 | 2 | 1 | 1 | 2 | 138 | 15.1 | 5.87 |
| A0A1Q3EFH4 | ATP synthase F1 alpha subunit OS=Lentinula edodes OX=5353 GN=LENED_007868 PE=4 SV=1 - [A0A1Q3EFH4_LENED] | 4.63 | 3.57 | 3 | 1 | 1 | 2 | 364 | 39.9 | 8.29 |
| Q96WQ6 | Fruit body lectin OS=Pleurotus cornucopiae OX=5321 GN=PCL-F1 PE=4 SV=1 - [Q96WQ6_PLECO] | 4.11 | 7.64 | 1 | 1 | 1 | 2 | 144 | 16.2 | 7.34 |
| A0A0X8XLC2 | Subtilisin-like peptidase OS=Pleurotus pulmonarius OX=28995 GN=ppp1 PE=2 SV=1 - [A0A0X8XLC2_PLEPU] | 3.56 | 4.15 | 2 | 1 | 1 | 1 | 386 | 38.7 | 8.21 |
| P81055 | Peptidyl-Lys metalloendopeptidase OS=Pleurotus ostreatus OX=5322 GN=MEP PE=1 SV=1 - [PLMP_PLEOS] | 3.16 | 7.74 | 1 | 1 | 1 | 1 | 168 | 17.9 | 6.24 |
| A0A1Q3ER18 | S-adenosylmethionine synthase OS=Lentinula edodes OX=5353 GN=LENED_011822 PE=3 SV=1 - [A0A1Q3ER18_LENED] | 3.12 | 3.83 | 3 | 1 | 1 | 1 | 392 | 43.0 | 6.25 |
| K5XND7 | Uncharacterized protein OS=Agaricus bisporus var. burnettii (strain JB137-S8 / ATCC MYA-4627 / FGSC 10392) OX=597362 GN=AGABI1DRAFT_116041 PE=4 SV=1 - [K5XND7_AGABU] | 2.91 | 2.53 | 4 | 1 | 1 | 1 | 396 | 45.1 | 5.25 |
| A0A151VW39 | Heat shock protein sks2 OS=Hypsizygus marmoreus OX=39966 GN=sks2 PE=3 SV=1 - [A0A151VW39_HYPMA] | 2.65 | 2.61 | 3 | 1 | 1 | 1 | 612 | 66.8 | 5.67 |
| A0A151W680 | Uncharacterized protein OS=Hypsizygus marmoreus OX=39966 GN=Hypma_02877 PE=4 SV=1 - [A0A151W680_HYPMA] | 0.00 | 1.06 | 1 | 1 | 1 | 1 | 565 | 61.9 | 9.33 |
