# Supplementary figures and images for: In Vitro Hepatoprotective and Human Gut Microbiota Modulation of Polysaccharide-Peptides in Pleurotus citrinopileatus
Source: Front Cell Infect Microbiol. 2022 May 20;12:892049. doi: 10.3389/fcimb.2022.892049 (PMC9165600; doi:10.3389/fcimb.2022.892049)

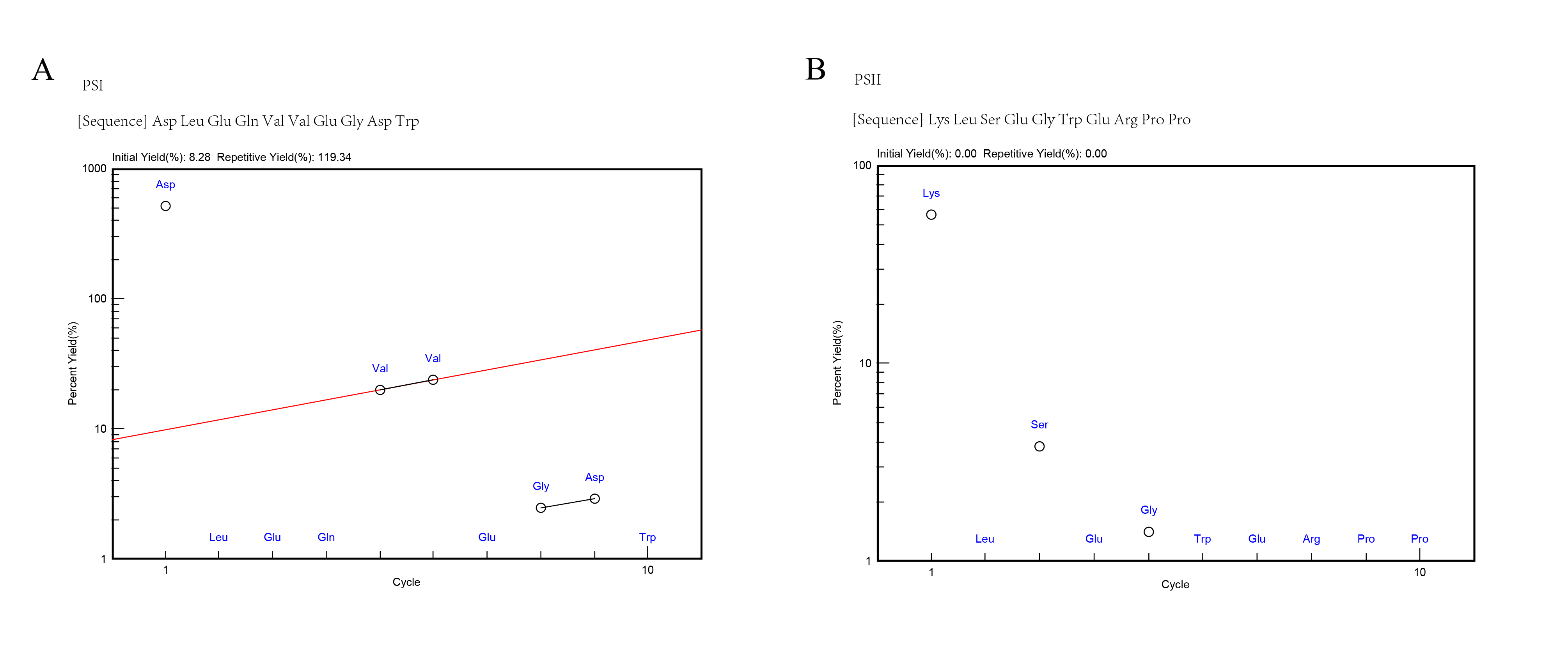

Supplement: Supplementary file 1 [file DataSheet_1.zip › Image 1.TIF]
